# Antidementia drug treatment in dementia networks in Germany: use rates and factors associated with treatment use

**DOI:** 10.1186/s12913-015-0855-7

**Published:** 2015-05-22

**Authors:** Markus Wübbeler, Diana Wucherer, Johannes Hertel, Bernhard Michalowsky, Steffen Heinrich, Saskia Meyer, Susanne Schaefer-Walkmann, Wolfgang Hoffmann, Jochen René Thyrian

**Affiliations:** German Center for Neurodegenerative Diseases (DZNE), Research Group “Interventional Health Care Research”, Site Rostock/Greifswald, Ellernholzstr. 1-2, Greifswald, D-17487 Germany; German Center for Neurodegenerative Diseases (DZNE), Site Rostock/ Greifswald, Ellernholzstr. 1-2, Greifswald, D-17487 Germany; German Center for Neurodegenerative Diseases (DZNE), Research Group “Care Structures” Site Witten, Stockumer Str. 12, Witten, D-58453 Germany; Department of Human and Health Science, University of Bremen, Grazer Str. 4, Bremen, D-28359 Germany; Institute for Community Medicine, Section Epidemiology of Health Care and Community Health, Ernst-Moritz-Arndt-University Greifswald, Ellernholzstr. 1-2, Greifswald, D-17487 Germany; Institute for Applied Social Sciences, Rotebühlstr. 131, Stuttgart, D-70197 Germany

**Keywords:** Dementia, Networks, Antidementia, Integrated care, Collaborative care

## Abstract

**Background:**

Dementia networks in Germany constitute a specialised setting for integrated dementia care and have shown benefits on relevant outcomes, including those of drug treatment. National guidelines recommend treatment with acetylcholinesterase inhibitors (donepezil, galantamine, rivastigmine) or the N-Methyl-D-Aspartate antagonist (memantine) to reduce cognitive symptoms. However, prescription rates are lower than 30 % in general practises. This study aims to describe antidementia drug treatment and the factors that are associated with the treatment in different dementia networks across Germany.

**Methods:**

We have analysed the socio-demographic, clinical and utilisation data from 560 patients with dementia (PWD), as well as data from their caregivers, in 13 selected dementia networks in Germany. The patients and caregivers were interviewed in their homes or in the network facilities. Multiple logistic regression models were fitted to evaluate the socio-demographic and clinical factors associated with the utilisation of antidementia drug treatment in the various networks.

**Results:**

In all of the networks in the study, 52 % of the participants received an antidementia drug treatment. Factors associated with the utilisation of the antidementia drug treatment were: formal diagnosis (OR = 16.81, p < 0.001), association with a physician in the network (OR = 3.69, p < 0.001), higher number of comorbidities (OR = 0.88, p = 0.039), living alone (OR = 0.51, p = 0.032) and higher age (OR = 2.97, p = 0.002).

**Conclusion:**

Medical treatment of PWD with antidementia drugs in dementia networks in Germany is more frequent than in primary and nursing home care settings. Our findings also suggest that participants with a formal diagnosis and a physician in the network have increased rates of receiving antidementia drug treatments. These findings suggest that dementia networks focusing on medical treatment should support diagnostic procedures and incorporate physician specialists.

## Background

According to Prince *et al.* [1], it was estimated that 35.6 million people worldwide were suffering from dementia in 2010. In Germany approximately 1.5 million people are affected by dementia [2]. Dementia is one of the most challenging global problems, especially for aging societies such as the one in Germany. In Germany, the appropriate treatment and care of people with dementia (PWD) has been declared a public health priority. However, appropriate PWD treatment requires a specialised mental health care structure [3]. Among professionals, there is agreement about the advantages of an integrated multi-modal care approach to address the specific needs of dementia care [4–6]. Dementia networks (DN) in Germany may be considered as a model for such integrated care. DN have shown potential on relevant outcomes such as pharmacological dementia treatment [7]. Even though DN are composed quite differently, they share a common goal: providing adequate treatment to their users. Such care includes early diagnosis, integrated treatment, reduction of caregiver burden, and the provision and dissemination of information concerning dementia disease [8]. Thus, DN address the transnational health policies of the European Union Council and are an important setting for dementia care [9]. To better understand DN a typical example is as follows:

The network is built by general practitioners and specialized neurologist/ psychiatrist in residency, hospitals, medical and social institutions. The network provides an interdisciplinary, cooperative model incorporating various medical care disciplines. The DN addresses two main goals: first, an early and differential formal diagnosis and second, a person-centered and comprehensive therapy according to the progression of the syndrome. To achieve these goals, DN established a specific dementia care pathway.

Antidementia drugs are currently considered the primary medical treatment for dementia [10]. The national guidelines in Germany recommend substances that include acetylcholinesterase inhibitors and the N-Methyl-D-Aspartate antagonist (memantine) [10, 11]. Donepezil, galantamine and rivastigmine are approved for the treatment of mild to moderate Alzheimer’s dementia and memantine for the treatment of moderate to severe Alzheimer’s dementia and although the effects are inconsistent. Ginkgo is approved but not recommended in the national guidelines for the treatment of dementia [10, 12]. Rivastigmine is the approved treatment for mild to moderate dementia in Parkinson’s disease.

Van den Bussche *et al.* [13] analysed antidementia prescription rates in a nationwide statutory health insurance company study of 1,848 PWD and found that 27 % of the group had at least one antidementia prescription. Furthermore, PWD with a formal diagnosis of Alzheimer (35 %) received more prescriptions than those with an unspecific type of diagnosis (7 %) or with a diagnosis of vascular dementia (7 %). If PWD were treated by a specialist, they received more antidementia prescriptions compared to those treated by a principal care physician (PCP) [13]. In Germany, PCP are considered as the gateway and point of entry to a medical treatment. They are responsible for treatment in general. In specialised settings such as German nursing homes, Huber *et al.* [14] found that 15.2 % of 8,017 PWD were taking at least one antidementia drug. Furthermore, in a statutory health insurance study based on general population data, Hoffmann *et al.* [15] found that age is a predictor for increased rates of antidementia drug treatment. In a register based study, Taipale *et al.* [16] observed that 84 % of the 6,858 community-dwelling PWD were being treated with at least one antidementia drug. A study in France by Tifratene *et al.* [17] showed that 76.9 % of the 26,809 individuals who were registered in the French National Alzheimer’s Bank and had received a diagnosis were treated with antidementia drugs.

However, little is known about treatment with antidementia drugs in specialised settings such as DN. Therefore, the objectives of the present analysis are to describe antidementia drug treatment and to analyse the factors that influence treatment in the specialised setting of DN in Germany.

## Methods

### Sample and data collection

The analysis is based on cross-sectional data of the ongoing study “Dementia Networks in Germany (DemNet-D)”. DemNet-D was conducted to analyse structures, procedures, and outcomes of DN in Germany using qualitative and quantitative methods. Ethical approval was obtained from the Committee of Ethics at the University of Greifswald (register number BB 107/12). Thirteen DN throughout Germany were included in the study. These networks applied for funding to participate and were chosen by the funding agency—the Federal Ministry of Health (BMG). Inclusion criteria were (a) having been previously evaluated and (b) being considered a sustainable network. Currently the total number of DN in Germany is unknown, however the funding opportunity was made public and all existing DN in Germany were eligible to participate.

For the analyses, we collected data from 560 pairs of PWD and their caregivers. Inclusion criteria for each pair were (a) being served by one of the 13 networks and (b) issuing of written informed consent by both the PWD and the caregiver. Participants were randomly selected by the DN according to these inclusion criteria during a timeframe of 6 months. Data were collected through in-person interviews and paper questionnaires by trained interviewers. The interviewer were employed by the DN, the professional background was heterogeneous. All were experienced in communicating and dealing with PWD and caregivers by employment criteria. Specific qualification for conducting the assessments was provided by two group trainings and a written manual, both specifically designed for this study. The interview period lasted from the 1^st^ of February until the end of September 2013, and recruitment was conducted by regional DN staff. Except for the geriatric depression scale (GDS) [18] all information was given by the interviewed caregiver. Information of the GDS was based on the answers given by the PWD.

### Measures

The collected socio-demographic information included gender, age, living situation and region. Living situation was categorised as either (a) living alone in own household or (b) living together with others. Type of region was classified as either urban/suburban or rural. Socio-economic status (SES) was operationalized using the Scheuch-Winkler index [19], a combination of household income, years of education and profession and categorised as high, middle and low SES. Information about comorbidity was collected using a paper questionnaire. To identify which diseases affect each PWD, participants were provided with a list of the most common diseases in geriatrics (hypertension, hyperlipidemia, adipositas, diabetes, coronary heart disease, heart attack, cardiac insufficiency, stroke, asthma, chronic bronchitis, renal insufficiency, hepatic insufficiency, enteritis, gastritis, stomach ulcer, duodenal ulcer, arthrosis, rheumatoide arthritis, osteoporosis, chronic back pain, cancer, deafness, visual impairment) from that they could check off. More specific and rare diseases could be indicated in open text fields. Diagnoses were transferred into ICD 10 codes. Functional status was measured using the Instrumental Activities of Daily Living (IADL) score according to Lawton and Brody [20]. This index ranges from 8 (no activity restrictions in daily living) to 0 (comprehensive activity restrictions in daily living). Depression was measured using the short-form geriatric depression scale (GDS) [18], with a score greater than 5 indicating depression. The caregiver was also asked about the presence of a diagnosis and the type of the dementia from the PWD.

The DN was categorised into either (a) physician associated networks, or networks led by a specialist (neurologist/psychiatrist) or (b) others. The other networks where community oriented networks focused on care providers. They aim to improve the management, evaluation and service integration for PWD in the sector of nursing care providers. To assess drug treatment, drugs were recorded by name, dose, and frequency of use. The following drugs were considered: donepezil (N06AD02), rivastigmine (N06AD03), galantamine (N06AD04), memantine (N06AX01), and ginkgo biloba (N06DP01) [21].

We assessed the daily target dose according to the national guidelines and recommendations for acetylcholinesterase inhibitors and N-Methyl-D-Aspartate [10]. The daily dosage was categorised as “target dose as recommended” or “others,” including: 20 mg/d for memantine, 10 mg/d for donepezil, 6–12 mg/d (9.5 mg/d – transdermal therapeutic system) for rivastigmine, and 16–24 mg/d for galantamine [10]. We defined the period of intake as the beginning of the antidementia drug treatment until time of the interview.

### Statistical analysis

We used descriptive statistics to summarize the demographic characteristics of the sample. To evaluate the associations of the prescription of antidementia drugs, we performed multiple logistic regression analyses with the prescription of the antidementia drug as the outcome variable. The model was adjusted for age, sex, comorbidities, functional status, depression, living situation (dichotomous: alone vs. not alone), diagnosis of dementia, and the network association (medical vs. others). Additionally, we accounted for the correlated nature of our data because observations from PWD in the same network were unlikely to be independent. Thus, we included the network as a random effect in our model. Prior to fitting the final regression model, we checked for non-linear associations of the covariates with the outcome by using the multivariate fractional polynomial approach [22]. As expected, the association with age showed a departure from linearity and age was therefore categorised into four age groups using the quartiles of the birth years as cut-off values (group 1: 1910–1927; group 2: 1928–1932; group 3: 1933–1937; group 4: 1938–1969). For graphical analysis, we modelled age with restricted cubic splines using three equally spaced knots [23].

We listed the name of the drug and the prevalence of usage by the PWD in the cohort. To show the drug dosage, we used the mean frequencies of the intake per day and the dose of each antidementia drug in the cohort. Two-sided p-values were calculated with a two-sided significance level (p-value = 0.05). The statistical package used for the analysis was STATA 11 (StataCorp LP, Texas, USA).

## Results

Descriptive data are presented in Table 1. The majority of the participants were female (58.3 %). Only a low percentage of the PWD were not diagnosed with any type of dementia or did not know (n = 48, 8.7 %). Overall, 18.1 % lived in rural regions, and 21.3 % stated that they were living alone in their own household. The mean number of comorbidities was 3.9. Common diseases that were found in the study population were cardiovascular (n = 420, 80.6 %), with a total of 755 ICD 10 diagnoses, and orthopaedic (n = 301, 57.7 %), with a total of 441 ICD 10 diagnoses. Most of the participants were assigned to a low (60.6 %) or middle (29.6 %) socioeconomic status. In addition, 39.9 % (n = 196) showed depressive symptoms according to the GDS.

**Table 1:** Descriptive analysis of the study sample

	n*	*Variable*		
Age	555	years	m (SD)	79,7 (8.4)
Gender	557	female	%	325 (58.3)
Social Status	409	lower class	%	248 (60.6)
		middle class	%	121 (29.6)
		higher class	%	40 (9.8)
Formal Diagnosis of Dementia	551	yes	%	503 (91.3)
Type of Dementia	476			
Alzheimer Dementia	211	yes	%	44.3
Vascular Dementia	92	yes	%	19.3
Unspecific	155	yes	%	32.6
Others	18	yes	%	3.8
Antidementia Treatment	541	yes	%	283 (52.3)
Combination Therapy	283	yes	%	19 (6.7)
Region	553	rural	%	100 (18.1)
Living Situation	558	alone in own household	%	119 (21.3)
Functional Status	511	IADL score	median (range)	2 (0–8)
Depression	491	GDS	median (range)	4 (0–15)
Comorbidities	521	number of morbidity’s	mean (SD)	3.9 (2.1)
Consultation Specialists	532	treated by neurologist/psychiatrists	%	395 (74.2)
	533	visits during the last 6 months	mean (SD)	2.3 (1.5)
Consultation PCP	550	treated by primary care physicians (PCP)	%	513 (93.3)
	530	visits during the last 6 months	mean (SD)	3.9 (2.5)

*cases with missing values on the respective variable were excluded from calculation of frequencies and means; PCP included general practitioners and internists in primary practice; IADL instrumental activities of daily living (0- no function, 8- complete function), GDS geriatric depression scale short- form (0- no depression, 5- suspicion of depression), Type of dementia – Others (Lewy-Body-Dementia, Korsakov, Frontotemporal dementia)

### Antidementia drug treatment

Overall, half of the participants were treated with antidementia drugs (52.3 %, n = 283). Of these drugs, the most widely used were the acetylcholinesterase inhibitors (62.9 %, n = 168). Memantine was used by 37.1 % of the PWD (n = 99). Overall, 6.7 % (n = 19) of those PWD who were using antidementia drug therapy were treated with two antidementia drugs. The most common combinations were memantine/donepezil (n = 9) and rivastigmine/memantine (n = 5). In the study sample, 5.1 % (n = 29) of the PWD used ginkgo biloba substances: 13 PWD used an antidementia drug plus ginkgo biloba, and 15 PWD used the ginkgo biloba extract alone. In addition, 9.3 % (n = 25) of the PWD were treated with dosages other than the recommended target dosage according to the national guidelines [10] (Table 2).

**Table 2:** Antidementia drugs in the sample

Class	Number	Months of intake - mean (SD)	Dosage per day - (n)
Antidementia	*n = 267 (%)*		
1. Memantine	99 (37.1)	25.25 (25.1) / n = 77	20 mg/d (90)
2. Donepezil	85 (31.8)	24.72 (22.5) / n = 65	10 mg/d (79)
3. Rivastigmine	52 (19.5)	21.33 (19.9) / n = 40	9.5 mg/d (35)
4. Galantamine	31 (11.6)	38.14 (26.2) / n = 21	16 mg/d (29)
Ginkgo biloba			
1. Ginkgo	n = 29 (5.1)	24.24 (26.1) / n = 17	120 mg/d (23)

### Associations between socio-demographic and clinical variables and the use of anti-dementia drug treatment

**Fig. 1:**
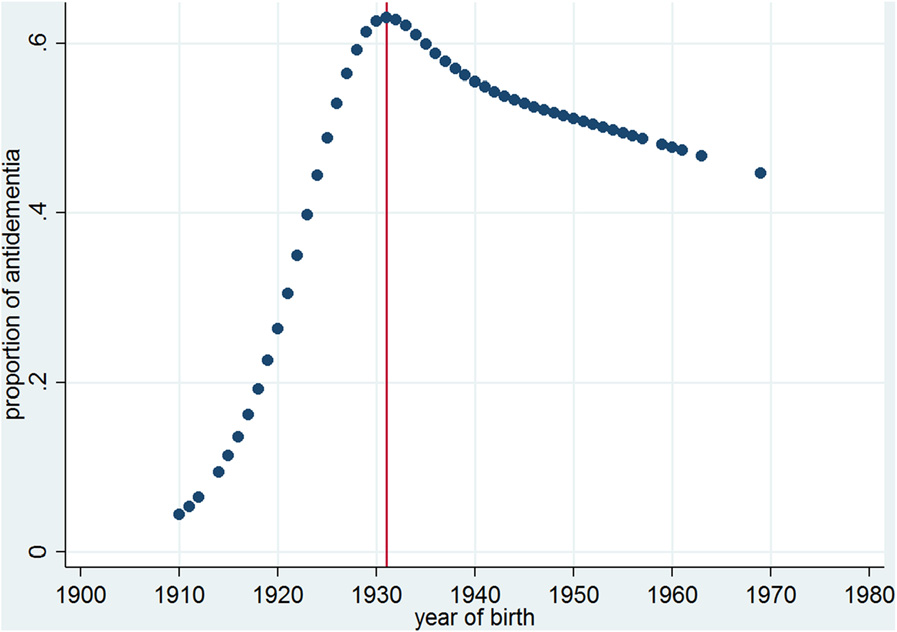
Proportion of antidementia treatment by year of birth

The regression model indicates that age, diagnosis, presence of a physician specialist in the network, number of comorbidities and living situation are associated with antidementia drug treatment. We found nonlinear associations for age. Although the second age group showed an odds ratio of 2.97 (p = 0.002) for antidementia treatment compared with the oldest age group, the third and the fourth age groups were not significantly different from the oldest age group. This result is reflected in Fig. 1, where the rate of antidementia drug treatment follows a reversed u-shaped curve.

We also found a significant association with the presence of a dementia diagnosis (p = <0.001) and antidementia treatment. The odds ratios for antidementia treatment of PWD with diagnosed dementia were 16.81 compared to the group without a formal diagnose. The largest antidementia drug treated group were PWD with a diagnosis of Alzheimer’s disease (73 %, n = 152). We found a difference of 32 % lower rates of antidementia treatment for the group of unspecified dementia diagnosis (41 %, n = 62). The physician associated network was a significant positive factor in influencing treatment with antidementia drugs (OR 3.69, p < 0.001). A higher number of comorbidities was associated with lower rates of antidementia drug treatment (OR 0.87, p = 0.039). Another significant factor in the regression model was the individual living situation. PWD who lived alone in their own households had a lower chance of using antidementia drug treatment (OR 0.51, p = 0.032) as compared to those living together with others (Table 3).

**Table 3:** Logistic regression models for prediction antidementia treatment

	Logistic regression			
Variables	(Treatment Antidementia)			
	OR	95 % CI		*p*-value
Gender (fem.)	0.915	0.550	1.521	0.733
Formal diagnosis	16.813	5.228	54.066	<0.001
Age (category)				
reference group: 1910-1927				
2 (1928–1932)	2.974	1.503	5.883	0.002
3 (1933–1937)	1.771	0.884	3.549	0.107
4 (1938–1969)	0.962	0.467	1.982	0.917
Living alone	0.510	0.275	0.945	0.032
Comorbidities	0.879	0.778	0.993	0.039
Depression	0.989	0.917	1.067	0.789
Functional Status	1.065	0.920	1.233	0.393
Physician associated	3.692	1.808	7.539	<0.001

Comorbidity; score of morbidities exclusive dementia disease, functional sta.; IADL instrumental activities of daily living (0- no function, 8- complete function), depression; geriatric depression scale short- form (0- no depression, 5- suspicion of depression)

## Discussion

Approximately half of the PWD (52 %) served in DN in Germany received an antidementia drug treatment. Koehler *et al.* [7] reported comparable numbers in their evaluation of a rural dementia network, in which 50.5 % of PWD received antidementia drugs. These numbers are higher than those estimated based on the national health insurance data. Van den Bussche *et al.* [13] reported 27 % for PWD in primary care, and Huber *et al.* [24] found a rate of only 15.2 % for nursing home residents in Germany. Other published health claims data show that 28 % of all PWD in Germany are treated with antidementia drugs (estimated from at least 1 million PWD) [25]. Utilisation rates of antidementia drugs are therefore 24-37 % higher in our sample of PWD in DN compared to population based data in Germany. Half of the participants (48 %) were not treated with antidementia drugs. Reasons for the non-antidementia treatment were not assessed but could be for example a missing indication (like for unspecific types or Lewy body and vascular forms of dementia). Antidementia drug treatment for these forms is only off-label (unspecific type) use or not-recommended. In our sample, 72 % of patients with Alzheimer’s disease were treated with antidementia drugs. Therefore PWD with Alzheimer type of dementia clearly receive higher numbers of antidementia drug treatment. Higher proportions with Alzheimer form were also found by Tifratene *et al.* [16]. Besides a lack of indication there also might be a group of PWD where the antidementia drug treatment has been already terminated. This could be related to late stages of dementia or the will of patients. However, unfortunately any information about the non-treatment is lacking. The responsible physician may provide additional information to assess the non-treatment in the sample. Further studies need to collect more information about the group of PWD not treated with antidementia drugs in DN.

In the group of PWD who received antidementia drugs, 6.7 % took combined antidementia drug therapy with two substances. Combination therapy has shown potential in delaying the deterioration of functional abilities in people with dementia [26]. Lopez *et al.* [27] found that users of combination therapy comprised 14.9 % of their sample. Similar percentages were found by Tifratene *et al.* [17] who reported 14.4 % of users of combination therapy in their nationwide sample. This finding is different from the results of our analysis, which showed less utilisation of combined antidementia drug treatments. However, this lower percentage may be related to the clinical guidelines in Germany, which describe a combination therapy only as an off-label prescription [10]. In addition, only 5 % of the PWD used ginkgo biloba drugs. This low percentage could be explained by the lack of international reviews that demonstrate the advantages of using ginkgo biloba for the treatment of dementia. Ginkgo biloba is not recommended by the pertinent national guidelines, though prescription rates have been found to be related to the physician’s specialisation [28]. In the observational study of Jeschke *et al.* [28], 67.6 % of the 577 PWD were treated with ginkgo biloba by a group of 22 physicians who specialise in complementary and alternative medicine. Given the low percentage of users of ginkgo biloba treatment in our study, it appears that health care providers within DN are following the national guidelines concerning antidementia drug treatment.

The treatment of dementia with antidementia drugs in PWD served by DN is associated with age, comorbidities, diagnosis, associated specialists in the network, number of comorbidities, and living situation. Utilisation of an antidementia drug treatment by PWD increased with age and decreased beyond the age of 82. This finding is contrary to the results of Hoffmann *et al.* who found that utilisation of antidementia drug treatment increased with age in the German population [15]. The authors evaluated the prescription rates of people older than 65 years and a diagnosis of dementia in data from a health insurance company in Germany. Our results suggest that reasons for the absence of antidementia drug treatment can be diverse, though health care providers should be aware of general treatment gaps for elderly PWD. Hoffmann *et al.* showed that the clinical condition is related to the prescription rates of acetylcholinesterase inhibitors [29]. They found a higher number of symptoms related to decreasing rates of antidementia drug treatment [29]. We also found that the number of comorbidities was associated with lower odds ratios for the prescription of antidementia drugs in our sample, but with a nonlinear association for increasing age. These data indicate that the number of comorbidities, clinical conditions, and age of the PWD are associated with lower rates of antidementia drug therapy. Prescriptions and medication adherence within a multi-morbidity population such as found in DN are dependent on organisational changes such as providing case management or enhancing multidisciplinary team work [30]. In our sample, we found that PWD who are living alone had reduced rates of antidementia drug treatment overall. This finding may be related to lower adherence or consultations with physicians. In a nationwide sample of 7,570 PWD living at home, Johnell *et al.* also found reduced rates for antidementia drug treatment [31]. An association between social environment and dementia care was also confirmed by Lehmann *et al.* in their sample of 349 community-dwelling PWD. Primary care physicians were less likely to detect dementia within subjects living alone [32]. An association between social environment and cognition has also been described. Fratiglioni *et al.* showed that number of contacts and quality of relationships are associated with cognition in PWD [33]. In addition to our results, these studies indicate the importance of the living situation of the PWD, calling for an awareness of PWD living alone. Health care networks like DN are more likely to integrate PWD living alone, as they integrate various health care providers who are aware of specific dementia health care concepts compared to a fragmented health care landscape [8].

The most relevant determinant for drug therapy was the presence of a dementia diagnosis. Although only 8.7 % of the PWD reported that they had not received a diagnosis, this factor was highly significant for antidementia drug treatment. This finding underscores the strategy of the World Health Organization, which promotes diagnosis as a key element for dementia care [6]. Without a specific diagnosis, PWD were also treated differently. Seventy-three percent of patients with Alzheimer’s disease received antidementia drugs, whereas only 41 % of patients with an unspecific dementia diagnosis were treated. However, approximately 9 % of the PWD stated a lack of a formal diagnose in their case. It is unclear if this group did not receive a formal diagnose or if they did not know. Furthermore, the specific diagnosis could differ between self-report and medical record. A validation of the formal diagnosis with the information documented by the responsible physician would provide additional information to this issue. However, this correlation was also confirmed by Hoffmann *et al.* [15]. The authors found that the chance for antidementia prescriptions was lower with the diagnosis of vascular dementia and higher with the diagnosis of Alzheimer disease [15].

In our data, we found that 11 % (n = 29) of the cholinesterase inhibitors and memantine was inconsistent with national guideline recommendations with respect to daily dose. Tifratene *et al.* found 20.7 % of antidementia pharmacotherapy to be non-compliant with national guidelines [17]. Therefore, discrepancies between guideline recommendations and utilisation data are common, based on the work of Tifratene *et al.* However, our definition of target dose would not detect non-compliance. We found no associations between function and signs of depression in our sample. Hoffmann *et al.* found relationships between care dependency status and lower rates of antidementia drug treatment [29]. The authors analysed cholinesterase inhibitor treatment without the drug class of Methyl-D-Aspartate antagonist (memantine). This association might be lower if memantine had been included. However, insignificant associations between function limitations, signs of depression and pharmaceutical antidementia drug treatment may be related to the integrated care structure of DN. Overall, the antidementia drug treatment figures of DN are clearly higher than the figures that have been published in Germany thus far.

## Conclusion

PWD using DN in Germany received antidementia drugs more frequently than PWD in primary or nursing home care situations. In these networks, factors that most influenced using antidementia drug treatments were formal diagnosis of dementia, presence of a physician in the network, number of comorbidities, living situation, and age. Based on these findings, further interventions should consider the effects of PWD living alone, prevent discrimination of the elderly and provide low threshold diagnostic services. Involvement of specialised physicians in DN would likely help to improve rates of antidementia drug treatment. Gaps between guideline recommendations and antidementia drug treatment should be examined more closely to eliminate risks of ineffective treatments and potential negative side effects.

### Limitations

The study has limitations that decrease the generalisability. Results are based on the self-report only. Data could not be verified with the PWDs primary physician or with health insurance information. Future research should collect additional information from the health insurance company and/ or the responsible physician. Furthermore, there could be a selection bias in the DN under examination. However, participation was open to all DN in Germany.

## References

[1] Prince M, Bryce R, Albanese E, Wimo A, Ribeiro W, Ferri CP (2013). The global prevalence of dementia: a systematic review and metaanalysis. Alzheimers Dement.

[2] Bickel H. Die Epidemiologie der Demenz [The epidemiology of dementia] In: Infoblatt Demenz. Deutsche Alzheimer Gesellschaft. 2014. https://www.deutsche-alzheimer.de/fileadmin/alz/pdf/factsheets/infoblatt1_haeufigkeit_demenzerkrankungen_dalzg.pdf Accessed 18 May 2015.

[3] Thornicroft G, Tansella M (2004). Components of a modern mental health service: a pragmatic balance of community and hospital care. Br J Psychiatry.

[4] Villars H, Oustric S, Andrieu S, Baeyens JP, Bernabei R, Brodaty H (2010). The primary care physician and Alzheimer’s disease: an International Position Paper. J Nutr Health Aging.

[5] Wolfs CAG, Kessels A, Dirksen CD, Severens JL, Verhey FRJ. Integrated multidisciplinary diagnostic approach for dementia care: randomised controlled trial. Br J Psychiatry 2008;300–5. 10.1192/bjp.bp.107.035204 18378994

[6] World Health Organization and Alzheimer’s Disease International. Dementia A Public Health Priority. In: Mental Health. World Health Organization. 2012. http://www.who.int/mental_health/publications/dementia_report_2012/en/. Accessed 20 May 2015.

[7] Köhler L, Meinke-Franze C, Hein J, Fendrich K, Heymann R, Thyrian JR (2014). Does an interdisciplinary network improve dementia care? Results from the IDemUck-study. Curr Alzheimer Res.

[8] Lemieux-Charles L, Chambers LW, Cockerill R, Jaglal S, Brazil K, Cohen C (2005). Evaluating the Effectiveness of Community-Based Dementia Care Networks: the Dementia Care Networks’ Study. Gerontologist.

[9] The Council of the European Union. Council Conclusions on public health strategies to combat neurodegenerative diseases associated with ageing and in particular Alzheimer’s disease. In: 2916th Employment, Social Policy, Health and Consumer Affairs Council Meeting. 2008. http://www.consilium.europa.eu/ueDocs/cms_Data/docs/pressData/en/lsa/104778.pdf. Accessed 20 May 2015.

[10] Deutsche Gesellschaft für Psychiatrie, Psychotherapie und Nervenheilkunde and Deutsche Gesellschaft für Neurologie. S3 - Leitlinie "Demenzen" [Guideline Dementia]. In: Diagnose und Behandlungsleitlinie Demenz. 2009. http://link.springer.com/chapter/10.1007/978-3-642-13092-2_2. Accessed 20 May 2015.

[11] Deutsche Gesellschaft für Allgemeinmedizin und Familienmedizin. Demenz [Dementia]. In: Leitlinien. Deutsche Gesellschaft für Allgemeinmedizin und Familienmedizin. 2008. http://www.degam.de/files/Inhalte/Leitlinien-Inhalte/Dokumente/DEGAM-S3-Leitlinien/LL-12_Langfassung_TJ_03_korr_01.pdf. Accessed 15 May 2015.

[12] Birks J, Evans GJ: Ginkgo biloba for cognitive impairment and dementia. Cochrane Library 2009; doi: 10.1002/14651858.CD003120.pub3. 10.1002/14651858.CD003120.pub3 PMC13076002 19160216

[13] van den Bussche H, Kaduszkiewicz H, Koller D, Eisele M, Steinmann S, Glaeske G (2011). Antidementia drug prescription sources and patterns after the diagnosis of dementia in Germany: results of a claims data-based 1-year follow-up. Int Clin Psychopharmacol.

[14] Huber M, Kolzsch M, Rapp MA, Wulff I, Kalinowski S, Bolbrinker J (2012). Antipsychotic drugs predominate in pharmacotherapy of nursing home residents with dementia. Pharmacopsychiatry.

[15] Hoffmann F, van den Bussche H, Glaeske G, Kaduszkiewicz H (2010). Eight-year prescription trends of memantine and cholinesterase inhibitors among persons 65 years and older in Germany. Int Clin Psychopharmacol.

[16] Taipale H, Transkanen A, Koponen M, Tolppanen A-M, Tiihonen J, Hartikainen S. Antidementia drug use among community-dwelling individuals with Alzheimer’s disease in Finland: a nationwide register-based study. Int Clin Psychopharmacol. 2014;29:216–23. 10.1097/YIC.0000000000000032 PMC4047310 24608822

[17] Tifratene K, Le Duff F, Pradier C, Quetel J, Lafay P, Schnück S (2012). Use of drug treatments for Alzheimer’s disease in France: a study on a national level based on the National Alzheimer’s Data Bank (Banque Nationale Alzheimer). Pharmacoepidemiol Drug Saf.

[18] Sheikh JI, Yesavage JA (1986). Geriatric Depression Scale (GDS): recent evidence and development of a shorter version. Clin Gerontol.

[19] Winkler J, Stolzenberg J. Der Sozialschichtindex im Bundesgesundheitssurvey [Social class index in the Federal Health Survey]. Das Gesundheitswesen. 1999;Sonderheft 2:178–83. 10726418

[20] Lawton MP, Brody EM (1969). Assessment of older people: self-maintaining and instrumental activities of daily living. The Gerontologist.

[21] Deutsches Institut für medizinische Dokumentation und Information (DIMDI). ATC-Klassifikation mit definierten Tagesdosen DDD [ATC-Classification with Defined Daily Doses]. In: Klassifikationen, Terminologien, Standards. 2013. https://www.dimdi.de/static/de/klassi/atcddd/index.htm. Accessed 20 May 2015.

[22] Royston P, Sauerbrei W (2008). Multivariable Model-Building - A pragmatic approach to regression analysis based on fractional polynomials for continuous variables.

[23] Harrell FE (2001). Regression Modeling Strategies. With Applications to Linear Models, Logistic Regression, and Survival Analysis.

[24] Huber M, Kölzsch M, Rapp MA, Wulff I, Kalinowski S, Bolbrinker J, et al. Antipsychotic Drugs Predominate in Pharmacotherapy of Nursing Home Residents with Dementia. Pharmacopsychiatry 2012; 45:182–8. 10.1055/s-0031-1301285 22430201

[25] Schwabe U (2013). Antidementiva [Antidementia]. Arzneiverordnungs-Report 2013.

[26] Atri A, Shaughnessy LW, Locascio JJ, Growdon JH (2008). Long-term course and effectiveness of combination therapy in Alzheimer disease. Alzheimer Dis Assoc Disord.

[27] Lopez OL, Becker JT, Wahed AS, Saxton J, Sweet RA, Wolk DA, et al. Long-term effects of the concomitant use of memantine with cholinesterase inhibition in Alzheimer disease. J Neurol Neurosurg Psychiatry. 2009;80:600–7. 10.1136/jnnp.2008.158964 PMC2823571 19204022

[28] Jeschke E, Ostermann T, Vollmar HC, Tabali M, Schad F, Matthes H (2011). Prescribing patterns in dementia: a multicentre observational study in a German network of CAM physicians. BMC Neurol.

[29] Hoffmann F, van den Bussche H, Wiese B, Schön G, Koller D, Eisele M (2011). Impact of geriatric comorbidity and polypharmacy on cholinesterase inhibitors prescribing in dementia. BMC Psychiatry.

[30] Smith SM, Soubhi H, Fortin M, Hudon C, O’Dowd T. Interventions for improving outcomes in patients with multimorbidity in primary care and community settings. The Cochrane Collaboration; 2012. doi:10.1002/14651858.CD006560.pub2. 10.1002/14651858.CD006560.pub2 22513941

[31] Johnell K, Religa D, Eriksdotter M. Differences in Drug Therapy between Dementia Disorders in the Swedish Dementia Registry: A Nationwide Study of over 7,000 Patients. Dement Geriatr Cogn Disord. 2013;35:239–48. 10.1159/000348408 23485654

[32] Lehmann SW, Black BS, Shore A, Kasper J, Rabins PV (2010). Living alone with dementia: lack of awareness adds to functional and cognitive vulnerabilities. Int Psychogeriatr.

[33] Fratiglioni L, Wang H-X, Ericsson K, Maytan M, Winblad B (2000). Influence of social network on occurrence of dementia: a community-based longitudinal study. Lancet.

